# Whole-Transcriptome Sequencing and ceRNA Network Analysis of the Hypothalamus During the Follicular Phase in High- and Low-Fecundity Xinjiang Goats

**DOI:** 10.3390/ijms27167330

**Published:** 2026-08-17

**Authors:** Yaqin Wu, Wenping Dong, Weiting Xing, Saidiguli Saimaiti, Yanli Xu, Min Zhang, Xuefeng Lv, Wenxin Zheng

**Affiliations:** 1 College of Life Science and Technology, Xinjiang University, Urumqi 830017, China; wuyaqin@stu.xju.edu.cn; 2 Xinjiang Uyghur Autonomous Region Academy of Animal Science, Urumqi 830011, China; 20242113019@stu.shzu.edu.cn (W.D.); 13899853128@163.com (W.X.); m13899942203_1@163.com (S.S.); xyl652701@163.com (Y.X.); 3 College of Animal Science and Technology, Shihezi University, Shihezi 832003, China; 4 College of Animal Science, Xinjiang Agricultural University, Urumqi 830052, China; summer_email1020@163.com

**Keywords:** HPG axis, neuroendocrine regulation, non-coding RNA, chi-miR-141, *PCDHB13*, reproductive performance

## Abstract

Fecundity affects herd expansion and economic efficiency in goats and is under the control of the hypothalamic–pituitary–gonadal (HPG) axis and intricate RNA regulatory networks. Herein, hypothalamic tissues from high- and low-fecundity Xinjiang goats during the follicular phase were analyzed using integrated mRNA/lncRNA transcriptome sequencing and small RNA sequencing, followed by differential expression, functional enrichment, and competing endogenous RNA (ceRNA) network analyses. Sixty-nine differentially expressed mRNAs, 202 differentially expressed lncRNAs, and 1 differentially expressed miRNA were identified between the two groups. Functional enrichment revealed that differentially expressed RNA-associated genes were mainly involved in hormone regulation and secretion; neuroactive ligand–receptor interactions; cAMP signaling; oxidative phosphorylation; the electron transport chain; and MAPK, TGF-β, and PI3K-Akt pathways. These findings link fecundity differences in Xinjiang goats to hypothalamic neuroendocrine signaling, mitochondrial energy metabolism, and post-transcriptional regulation during the follicular phase. ceRNA analysis suggested that 16 lncRNAs may be involved in the putative regulation of *PCDHB13* through chi-miR-141, highlighting five key candidate lncRNA–chi-miR-141–PCDHB13 axes. *PCDHB13*, a protocadherin involved in neuronal adhesion and neural circuit formation, suggests that the lncRNA–chi-miR-141–PCDHB13 module may influence hypothalamic signaling and reproductive regulation during the follicular phase. Overall, this study identifies candidate RNAs and ceRNA axes that underlie fecundity differences in Xinjiang goats.

## 1. Introduction

The goat (*Capra hircus*) is an important small-ruminant livestock species that contributes substantially to meat, milk, and fiber production, and is instrumental in regional livestock economies. Fecundity is a key economic trait that influences herd expansion and production efficiency, with litter size directly influencing productivity and breeding value in goat populations [1,2]. The Xinjiang goat is an important indigenous breed in northwestern China, characterized by strong environmental adaptability and considerable production potential. Prior research has revealed distinct hypothalamic gene expression patterns and serum levels of follicle-stimulating hormone (FSH) and luteinizing hormone (LH) during the estrous cycle between high- and low-fecundity Xinjiang goats. This suggests that neuroendocrine regulation may contribute to variation in reproductive performance [3,4].

Goat fecundity is a complex quantitative trait influenced by genetic background, follicular development, ovulation rate, embryo survival, endocrine status, and environmental factors. A number of genes associated with reproduction, such as *BMPR1B*, *BMP15*, *GDF9*, and *KISS1*, have received thorough investigation. Among these, the transforming growth factor-β (TGF-β) superfamily members *BMPR1B*, *BMP15*, and *GDF9* are closely associated with follicular development, ovulation rate, and litter size in small ruminants [2,5,6,7]. However, fecundity is not determined by a few major-effect genes alone, and the functions of candidate genes may vary across breeds, tissues, physiological stages, and genetic backgrounds. Recent research using genome-wide association studies, whole-genome resequencing, and transcriptome analyses has identified numerous candidate genes and regulatory pathways associated with litter size, ovarian function, and reproductive performance in goats [1,2,8,9]. These findings highlight the importance of investigating fecundity from the perspective of multilayered molecular regulatory networks.

Mammalian reproductive activity is tightly regulated by the hypothalamic–pituitary–gonadal (HPG) axis. As the central neuroendocrine regulator, the hypothalamus regulates pituitary secretion of LH and FSH through the pulsatile release of gonadotropin-releasing hormone (GnRH), thereby influencing follicular development, ovulation, and ovarian hormone production [10]. The follicular phase is characterized by active follicular growth, enhanced estrogen feedback, and intensified neuroendocrine regulation preceding ovulation. Estrogen-positive feedback and the generation of the GnRH/LH surge are essential for ovulation and normal reproductive function in females [11]. Hence, comparing the hypothalamic transcriptome of high- and low-fecundity Xinjiang goats during the follicular phase reduces estrous cycle stage-associated variation and helps reveal the upstream neuroendocrine mechanisms governing reproductive differences.

Advances in high-throughput sequencing technologies have provided powerful tools for elucidating the molecular basis of reproductive traits in goats. Previous transcriptomic studies of the goat hypothalamus have revealed significant differences in the expression profiles of messenger RNAs (mRNAs), long non-coding RNAs (lncRNAs), microRNAs (miRNAs), and circular RNAs (circRNAs) between animals with different reproductive capacities. These molecules are primarily involved in pathways related to GnRH and estrogen signaling, neuroactive ligand–receptor interaction, hormone secretion, and intracellular signal transduction [12,13,14,15]. In addition to protein-coding genes, non-coding RNAs have attracted increasing attention because of their regulatory roles in reproduction. miRNAs regulate gene expression by affecting the stability and translational efficiency of target mRNAs, whereas lncRNAs can influence gene expression through cis-, trans-, and post-transcriptional mechanisms. Furthermore, lncRNAs operate as competing endogenous RNAs (ceRNAs) via miRNA binding, thereby exerting indirect control over target mRNAs [16,17,18]. Previous studies demonstrated that non-coding RNAs participate in ovarian function, granulosa cell proliferation and apoptosis, steroidogenesis, and reproductive disorders [19,20]. In goats, mRNA–miRNA–lncRNA interactions and ceRNA networks have been widely used to investigate the regulatory mechanisms underlying fecundity, ovarian function, embryo implantation, and reproductive performance in the ovary, uterus, and oviduct [21,22,23].

Previous studies have examined individual RNA species and circRNAs in goat fecundity. However, comprehensive analyses of the coordinated regulation of mRNAs, lncRNAs, and miRNAs, together with ceRNA networks in the hypothalamus of Xinjiang goats during the follicular phase, are still limited. Therefore, in this study, hypothalamic tissues from high- and low-fecundity Xinjiang goats during the follicular phase were subjected to integrated mRNA/lncRNA transcriptome sequencing and small RNA sequencing. Differentially expressed mRNAs (DEmRNAs), lncRNAs, and miRNAs were identified, their functional characteristics were investigated through enrichment analyses, and candidate lncRNA–miRNA–mRNA regulatory networks were constructed. In this study, we identified candidate RNA molecules associated with fecundity differences in Xinjiang goats and provide a foundation for future functional validation and mechanistic studies of hypothalamic regulation of reproductive performance.

## 2. Results

### 2.1. Identification and Functional Analysis of DEmRNAs in the Hypothalamus of Xinjiang Goats with Different Fecundity Levels

Hypothalamic tissues from the high-fecundity (XJLDX) and low-fecundity (XJLSX) groups underwent RNA sequencing to identify DEmRNAs associated with reproductive performance. Quality assessment showed that the raw sequencing output per sample ranged from 17.07 to 19.61 Gb. All samples exhibited Q30 values above 95.70%, GC contents between 43.43 and 44.65%, and proportions of ambiguous bases (N) below 0.011% (Table S1). After quality filtering, 111.78–128.18 million clean reads were retained per sample, corresponding to 16.85–19.33 Gb of clean data. The retention rates of clean reads and clean data exceeded 98.71 and 98.56%, respectively, indicating excellent sequencing quality (Table S2).

**Figure 1:**
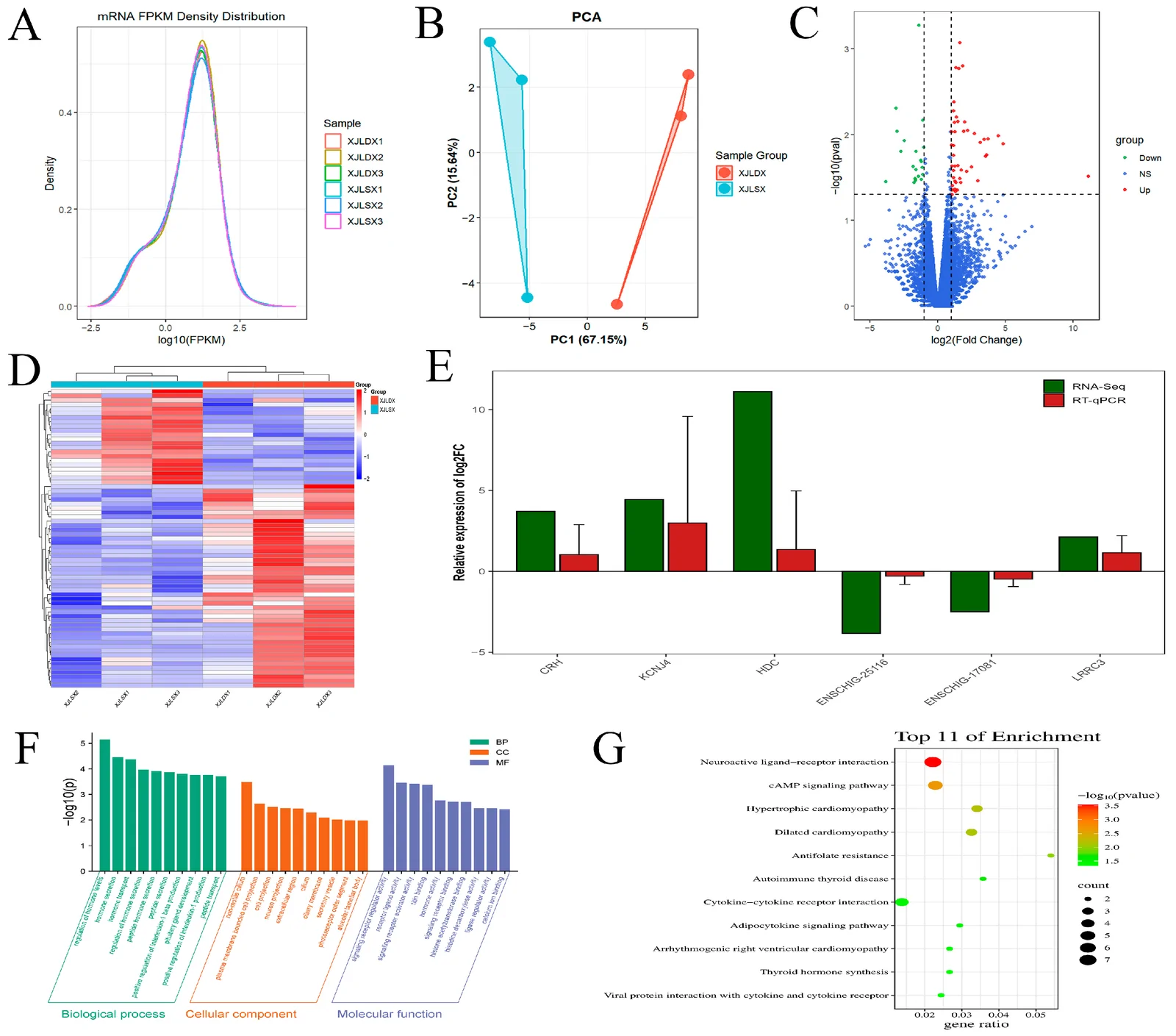
Identification and functional analysis of DEmRNAs between the XJLDX and XJLSX groups. (**A**) FPKM density distribution curves of mRNA expression levels across samples. (**B**) PCA of DEmRNAs. (**C**) Volcano plot of DEmRNAs. The *x*-axis represents log_2_FoldChange, and the *y*-axis represents −log_10_(*p*-value). (**D**) Hierarchical clustering heatmap of DEmRNA expression profiles. (**E**) Comparison of RNA-seq and RT-qPCR results. (**F**) GO enrichment analysis of DEmRNAs. The *x*-axis represents GO terms, and the *y*-axis represents −log_10_(*p*-value). (**G**) KEGG pathway enrichment analysis of DEmRNAs. DEmRNAs, differentially expressed mRNAs; FPKM, fragments per kilobase of transcripts per million mapped reads; GO, Gene Ontology; PCA, principal component analysis; XJLDX, high-fecundity group; XJLSX, low-fecundity group.

Alignment of clean reads to the reference genome yielded overall mapping rates of 97.05–97.79% and unique mapping rates of 93.11–95.42%. Genome annotation analysis showed that 64.25–65.84% of uniquely mapped reads were located within annotated gene regions, of which 45.45–56.13% mapped to exons (Table S3). Fragments per kilobase of transcripts per million mapped read (FPKM) density distribution curves demonstrated highly similar expression profiles across all samples (Figure 1A). Principal component analysis (PCA) revealed tight clustering of biological replicates within groups and clear separation between groups (Figure 1B), indicating good reproducibility and supporting subsequent differential expression analyses.

Using the criteria of *p* < 0.05 and |log_2_FC| > 1, 69 DEmRNAs were identified between the XJLDX and XJLSX groups (Table S4). Compared with those in the XJLDX group, expression of 47 genes was considerably upregulated and of 22 genes was downregulated in the XJLSX group (Figure 1C). Hierarchical clustering further demonstrated distinct group-specific expression patterns, with samples from each group clustering together and DEmRNA expression profiles corresponding closely to group classification (Figure 1D). To validate the RNA-seq data, six DEmRNAs were randomly selected for quantitative real-time PCR (RT-qPCR) analysis. The expression profiles obtained by RT-qPCR were in excellent accord with the sequencing outcomes (Figure 1E), further attesting to the dependability of the transcriptome data. Gene Ontology (GO) enrichment analysis revealed that the 69 DEmRNAs were significantly enriched in 669 GO terms (Figure 1F). The most significantly enriched terms in the biological process (BP) category were linked to hormone level regulation, hormone secretion, hormone transport, peptide hormone secretion, and pituitary gland development. In the cellular component (CC) category, enriched terms included cell projections, neuronal projections, extracellular regions, cilia, and secretory vesicles. In the molecular function (MF) category, significant enrichment was observed for hormone activity, receptor ligand activity, and signaling receptor activator activity. Kyoto Encyclopedia of Genes and Genomes (KEGG) pathway analysis revealed significant enrichment in neuroactive ligand–receptor interaction, the cAMP signaling pathway, and thyroid hormone synthesis (Figure 1G). Collectively, differential gene expression in the hypothalamus of Xinjiang goats with divergent fecundity is primarily associated with hormone secretion, neuroendocrine signaling, receptor–ligand interactions, and thyroid hormone-related regulatory processes.

### 2.2. Identification and Functional Analysis of Differentially Expressed lncRNAs (DElncRNAs) in the Hypothalamus of Xinjiang Goats with Different Fecundity Levels

**Figure 2:**
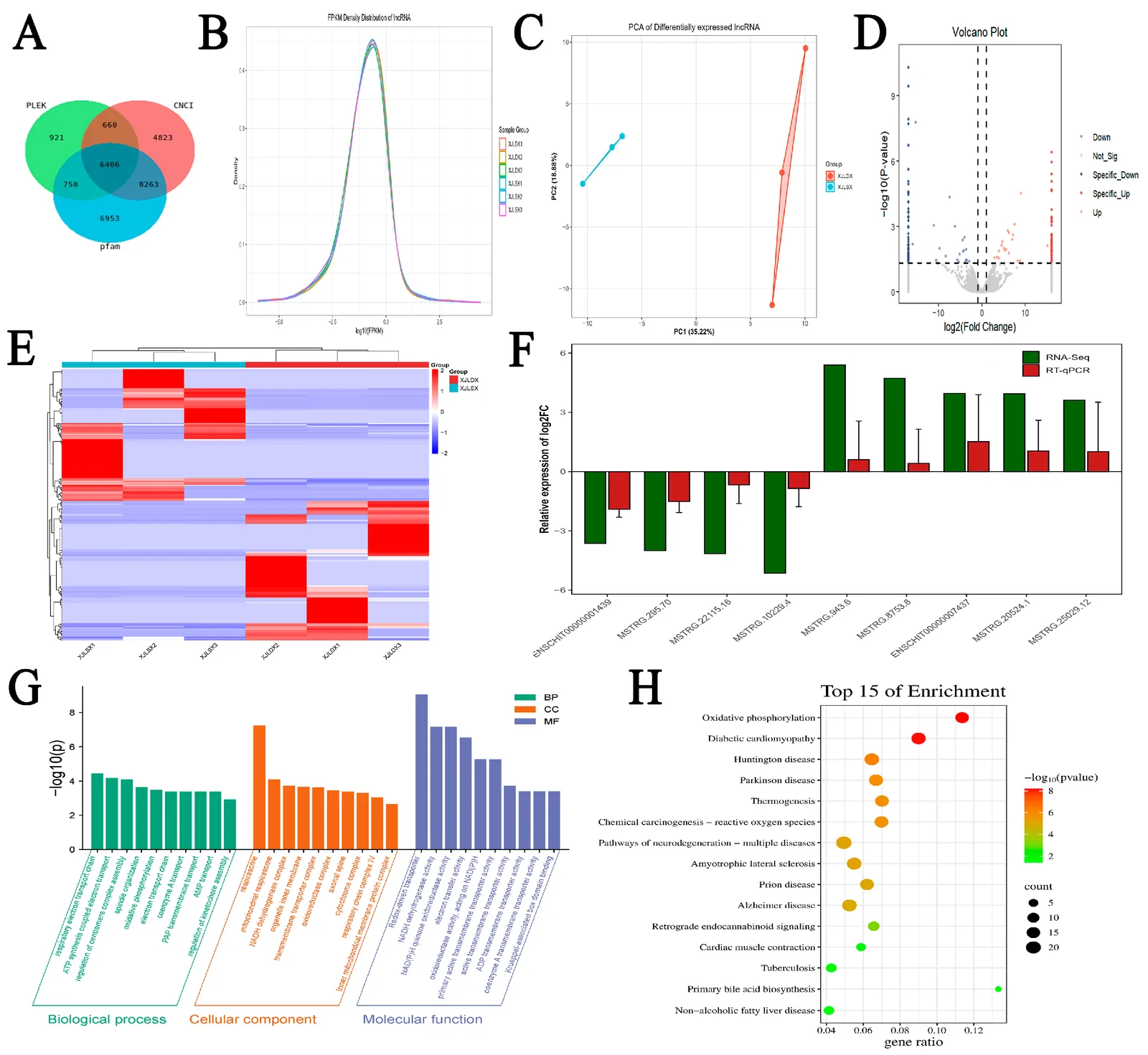
Identification and functional analysis of DElncRNAs between the XJLDX and XJLSX groups. (**A**) Venn diagram showing the coding potential prediction results of candidate lncRNAs identified using PLEK, CNCI, and Pfam. (**B**) FPKM density distribution curves of lncRNA expression levels across samples. (**C**) PCA of DElncRNAs. (**D**) Volcano plot of DElncRNAs. The *x*-axis represents log_2_FoldChange, and the *y*-axis represents −log_10_(*p*-value). (**E**) Hierarchical clustering heatmap of DElncRNA expression profiles. (**F**) Comparison between RNA-seq and RT-qPCR results. (**G**) GO enrichment analysis of DElncRNA target genes. The *x*-axis represents GO terms, and the *y*-axis represents −log_10_(*p*-value). (**H**) KEGG pathway enrichment analysis of DElncRNA target genes. XJLDX, high-fecundity group; XJLSX, low-fecundity group; DElncRNAs, differentially expressed lncRNAs; GO, Gene Ontology; KEGG, Kyoto Encyclopedia of Genes and Genomes; PCA, principal component analysis.

Following alignment of clean reads to the reference genome, transcript assembly was performed using StringTie v2.2.3, and candidate lncRNAs were identified using three coding-potential prediction tools: PLEK, CNCI, and Pfam (Figure 2A). In total, 6406 high-confidence transcripts lacking protein-coding potential were identified as lncRNAs. The FPKM density distribution curves showed substantial overlap among samples (Figure 2B). PCA demonstrated tight clustering of biological replicates within groups and clear separation between groups (Figure 2C), indicating good reproducibility.

Using the criteria of *p* < 0.05 and |log_2_FC| > 1, 202 DElncRNAs were identified between the XJLDX and XJLSX groups (Table S5). Compared with those in the XJLDX group, expression of 104 lncRNA was substantially upregulated and of 98 lncRNAs was significantly downregulated in the XJLSX group (Figure 2D). Hierarchical clustering analysis further showed distinct group-specific expression patterns, with samples from each group clustering together and DElncRNA expression profiles corresponding closely to the group classification (Figure 2E). To validate the sequencing results, nine DElncRNAs were randomly selected for RT-qPCR analysis. The expression patterns obtained using RT-qPCR were highly consistent with the RNA-seq data (Figure 2F), further confirming the reliability of the sequencing results.

To investigate the potential functions of DElncRNAs, GO and KEGG enrichment analyses were performed based on their predicted target genes. GO analysis revealed that DElncRNA target genes were primarily enriched in BPs associated with mitochondrial energy metabolism, oxidative phosphorylation, the electron transport chain, and transmembrane transport. Significant enrichment was also observed in cell division-related processes, including spindle organization, regulation of centromeric complex assembly, and kinetochore assembly (Figure 2G). KEGG pathway analysis showed significant enrichment in oxidative phosphorylation, thermogenesis, retrograde endocannabinoid signaling, cardiac muscle contraction, and primary bile acid biosynthesis pathways, with oxidative phosphorylation representing the most significantly enriched pathway (Figure 2H). Collectively, DElncRNAs associated with divergent fecundity may regulate mitochondrial function, energy metabolism, redox homeostasis, and neural signaling processes in the hypothalamus of Xinjiang goats.

### 2.3. Comparative Analysis of lncRNAs and mRNAs

**Figure 3:**
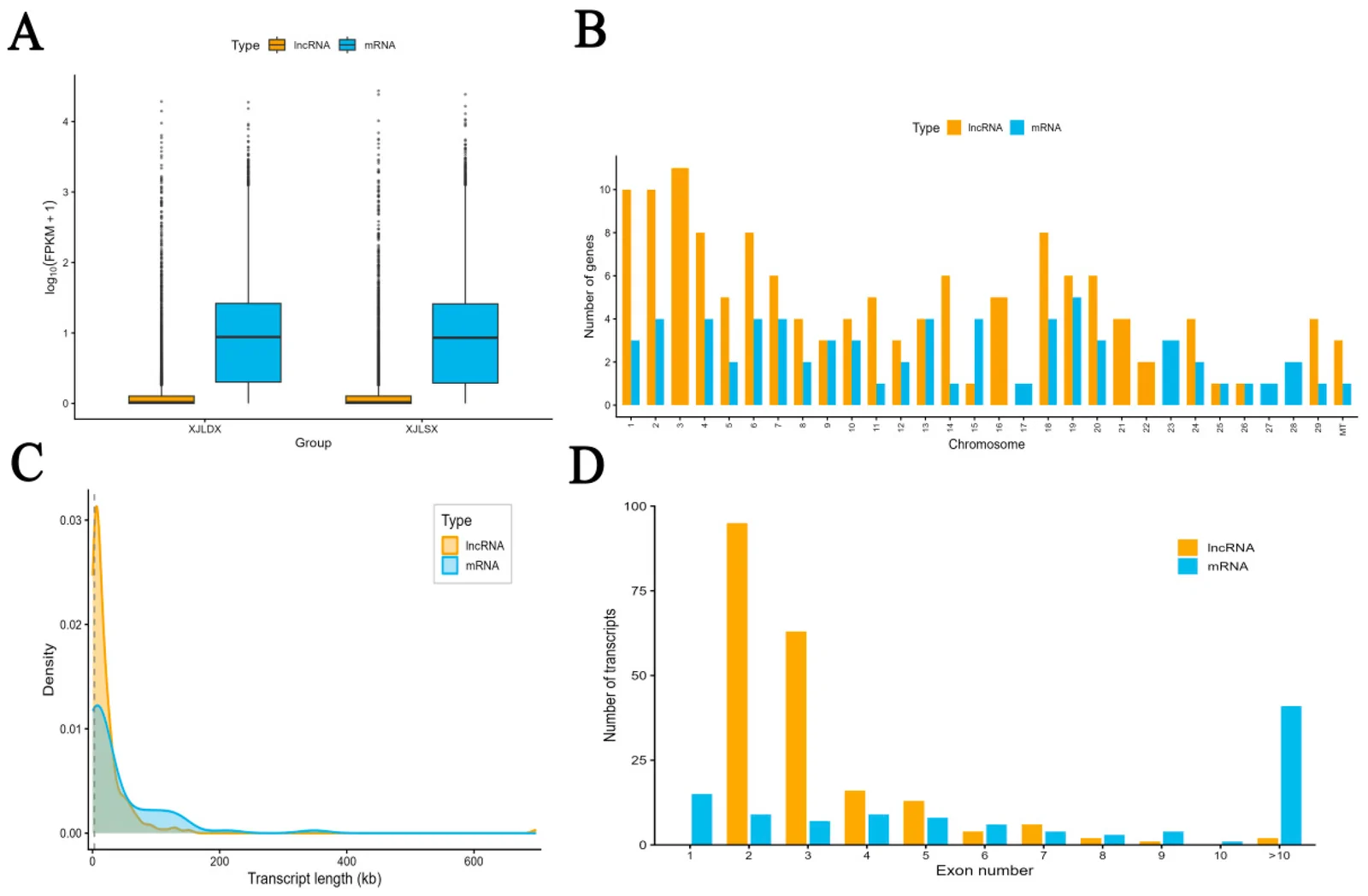
Comparative analysis of lncRNAs and mRNAs. (**A**) Boxplots showing the expression levels of lncRNAs and mRNAs in each group. Expression levels are presented as log_10_(FPKM + 1). (**B**) Chromosomal distribution of lncRNA and mRNA transcripts. (**C**) Comparison of transcript length distributions between lncRNAs and mRNAs. (**D**) Comparison of exon number distributions between lncRNAs and mRNAs. FPKM, fragments per kilobase of transcripts per million mapped reads.

To compare the expression patterns and structural characteristics of lncRNAs and mRNAs, their expression levels, chromosomal distributions, transcript lengths, and exon numbers were analyzed. The results showed that lncRNAs exhibited lower overall expression levels than those of mRNAs (Figure 3A). In contrast, lncRNA transcripts were more broadly distributed across chromosomes than were mRNA transcripts (Figure 3B). Analysis of transcript length distribution revealed that lncRNAs were generally shorter than mRNAs (Figure 3C). Furthermore, exon number analysis showed that most lncRNAs contained two to three exons, whereas mRNAs generally contained a greater number of exons (Figure 3D). These results indicate substantial differences between lncRNAs and mRNAs in both expression patterns and transcript structural characteristics.

### 2.4. Co-Expression Analysis of lncRNAs and mRNAs

**Figure 4:**
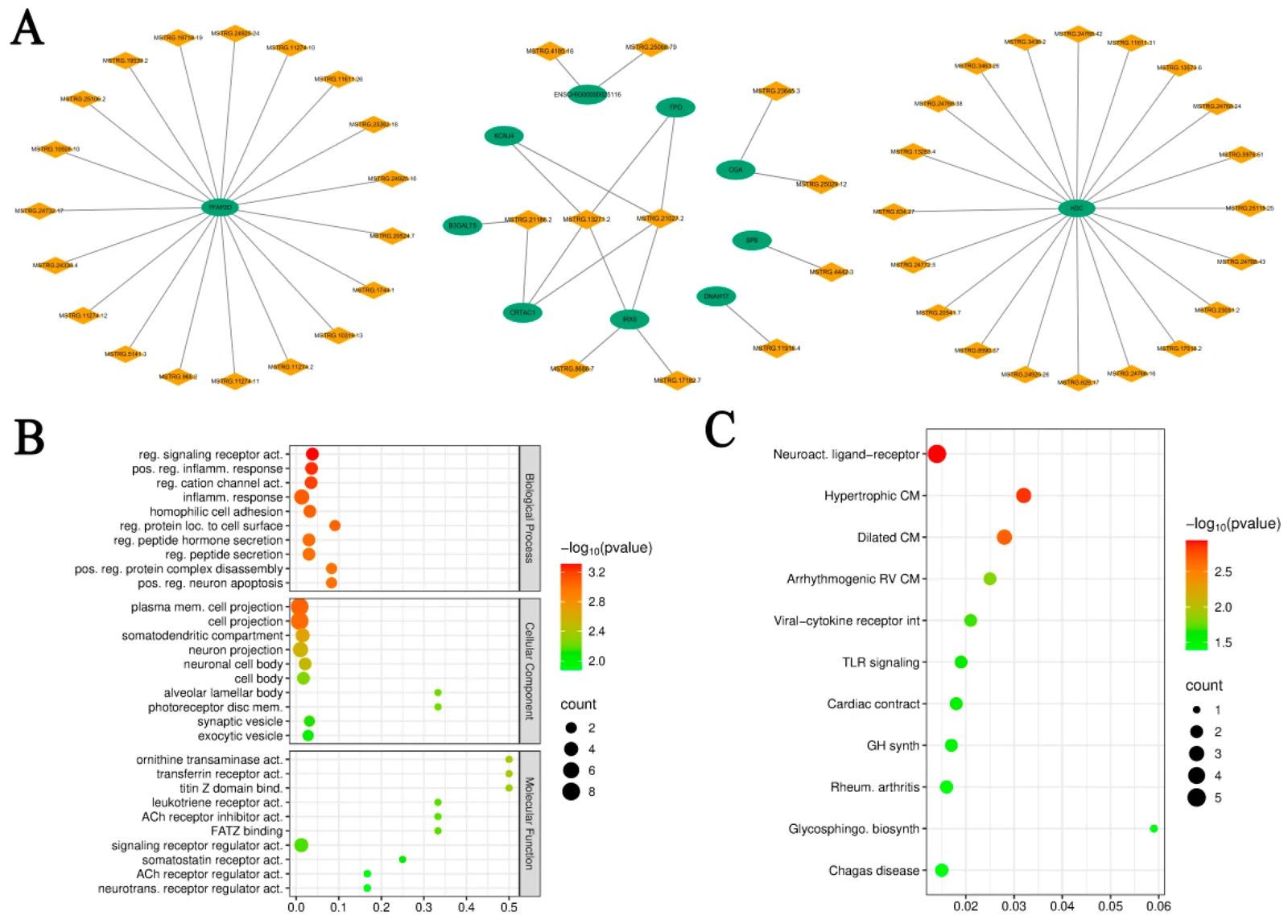
Co-expression analysis of lncRNAs and mRNAs. (**A**) Candidate lncRNA–mRNA co-expression network. Green ellipses represent mRNAs, and yellow diamonds represent lncRNAs. (**B**) GO enrichment analysis of mRNAs co-expressed with lncRNAs. The *y*-axis represents GO terms, and the *x*-axis represents enrichment scores. (**C**) KEGG pathway enrichment analysis of mRNAs co-expressed with lncRNAs. The *x*-axis represents enrichment scores, and the *y*-axis represents enriched pathways. GO, Gene Ontology; KEGG, Kyoto Encyclopedia of Genes and Genomes.

Pearson correlation analysis was performed using the expression profiles of DElncRNAs and DEmRNAs. Using the criteria of correlation coefficient > 0.95 and *p* < 0.05, 209 lncRNA–mRNA co-expression pairs were identified. After intersecting these pairs with the DEmRNA dataset, 175 potential DElncRNA–DEmRNA co-expression relationships were retained for subsequent analysis (Table S6). To highlight key regulatory relationships potentially associated with fecundity, representative lncRNA–mRNA pairs were selected based on correlation strength, differential expression levels, and functional annotation, and were visualized using Cytoscape v3.10.0 (Figure 4A).

Functional enrichment analysis of the co-expressed mRNAs, using Gene Ontology, identified significant enrichment in 299 biological processes, 31 cellular components, and 33 molecular function terms. The enriched GO terms were primarily associated with the regulation of peptide hormone secretion, positive regulation of neuronal apoptosis, cell projection organization, signaling receptor regulator activity, neurotransmitter receptor regulator activity, and hormone activity (Figure 4B). KEGG pathway enrichment analysis revealed that the co-expressed mRNAs were mainly associated with neuroactive ligand–receptor interaction, growth hormone synthesis, secretion and action, the Toll-like receptor signaling pathway, and cardiac muscle contraction (Figure 4C). These enrichment patterns were largely consistent with those observed for DEmRNAs, suggesting that lncRNAs may contribute to hypothalamic functional differences between high- and low-fecundity Xinjiang goats by regulating genes involved in neural signal transduction, hormone secretion, and receptor-mediated signaling pathways.

### 2.5. Identification and Functional Analysis of Differentially Expressed miRNAs (DEmiRNAs) in the Hypothalamus of Xinjiang Goats with Different Fecundity Levels

**Figure 5:**
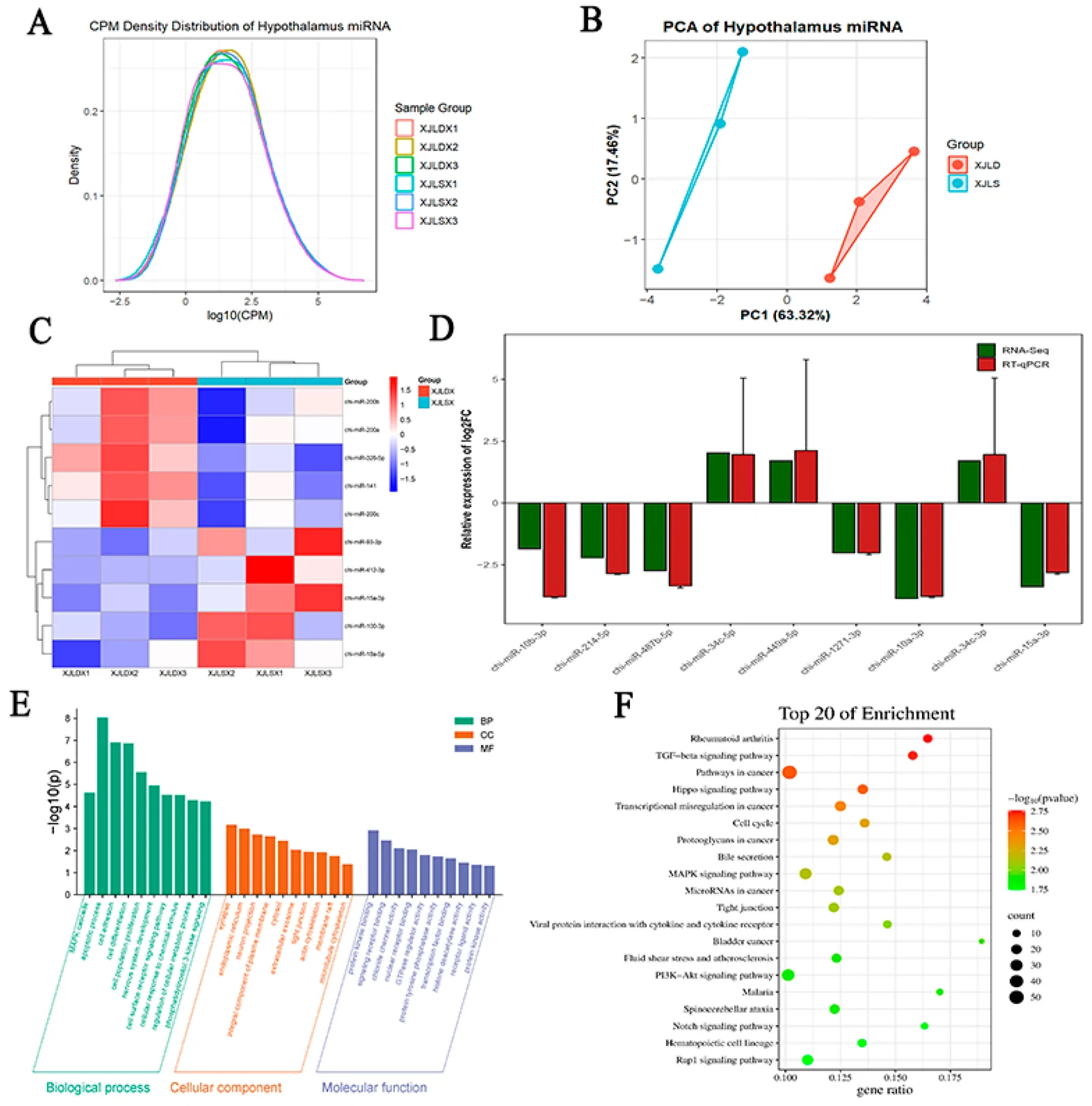
Identification and functional analysis of DEmiRNAs between the XJLDX and XJLSX groups. (**A**) CPM density distribution curves of miRNA expression levels across samples. (**B**) PCA of miRNA expression profiles. (**C**) Hierarchical clustering heatmap of miRNA expression profiles. (**D**) Comparison of RNA-seq and RT-qPCR results. (**E**) GO enrichment analysis of DEmiRNA target genes. The *x*-axis represents GO terms, and the *y*-axis represents −log_10_(*p*-value). (**F**) KEGG pathway enrichment analysis of DEmiRNA target genes. The *x*-axis represents enriched pathways, and the *y*-axis represents −log_10_(*p*-value). DEmiRNAs, differentially expressed miRNAs; XJLDX, high-fecundity group; XJLSX, low-fecundity group; CPM, counts per million; GO, Gene Ontology; KEGG, Kyoto Encyclopedia of Genes and Genomes; PCA, principal component analysis.

Hypothalamic tissues from the XJLDX and XJLSX groups were subjected to small RNA sequencing. After quality control, the valid reads obtained per sample ranged from 11.03 to 17.22 million, with 78.95–84.95% of the data retained. Alignment to the miRBase database, https://www.mirbase.org/ (accessed on 6 December 2025), identified 563–655 miRNAs per sample, including 400–415 known miRNAs and 163–240 novel miRNAs. The number of precursor miRNAs corresponding to known miRNAs ranged from 252 to 257. After removal of redundant sequences, 17,638–24,624 unique reads were retained, with total read counts ranging from 7.50 to 13.68 million (Table S7). The counts per million density distribution curves showed highly consistent miRNA expression profiles across samples (Figure 5A). PCA demonstrated tight clustering of biological replicates within groups and clear separation between groups (Figure 5B), indicating good reproducibility. Using the criteria of *p* < 0.05 and |log_2_FC| > 1, only one miRNA was identified as significantly upregulated in expression in the XJLSX group compared with those in the XJLDX group, whereas no significantly downregulated miRNAs with significantly downregulated expression were detected. Heatmap analysis showed highly similar expression patterns among biological replicates within each group and distinct expression differences between groups (Figure 5C). To validate the sequencing results, nine miRNAs, including the identified DEmiRNA, were selected for RT-qPCR analysis. The expression patterns obtained using RT-qPCR were largely consistent with the sequencing results (Figure 5D), confirming the reliability of the small RNA sequencing results.

To evaluate the potential functions of the DEmiRNA, a total of 1239 target genes were predicted (Table S8), followed by GO and KEGG enrichment analyses. GO analysis showed that the target genes were mainly enriched in biological processes such as apoptosis, cell adhesion, cell differentiation, the MAPK signaling cascade, cell population proliferation, and nervous system development. In the CC category, the enriched terms were mainly associated with synapses, neuronal projections, integral components of the plasma membrane, and extracellular exosomes. The enriched MF terms included protein kinase binding, signaling receptor binding, chloride channel activity, nuclear receptor binding, and GTPase regulator activity. KEGG pathway enrichment analysis showed significant enrichment in the TGF-β, MAPK, Hippo, and PI3K-Akt signaling pathways, as well as pathways related to the cell cycle and tight junctions. Collectively, the target genes of the DEmiRNA may be involved in hypothalamic cell proliferation and differentiation, nervous system development, intercellular communication, and multiple signal transduction pathways.

### 2.6. Construction of the mRNA–miRNA–lncRNA Regulatory Network

**Figure 6:**
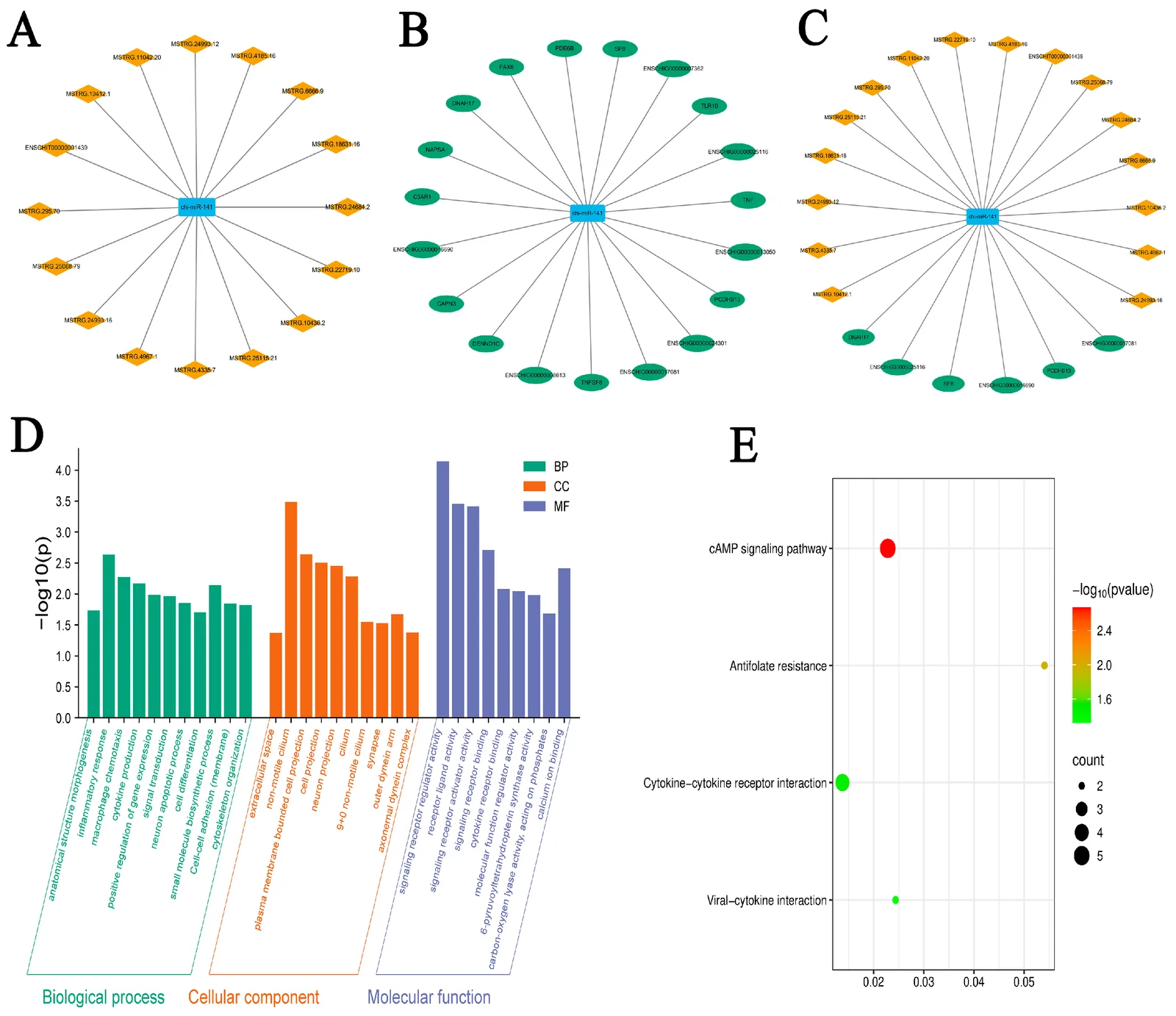
ceRNA interaction network analysis. (**A**) Potential miRNA–lncRNA interaction network. (**B**) Potential miRNA–mRNA interaction network. (**C**) Candidate lncRNA–miRNA–mRNA ceRNA network. Green ellipses represent mRNAs, blue rectangles represent miRNAs, and yellow diamonds represent lncRNAs. (**D**) GO enrichment analysis of mRNAs involved in the candidate ceRNA network. The *x*-axis represents GO terms, and the *y*-axis represents −log_10_(*p*-value). (**E**) KEGG pathway enrichment analysis of mRNAs involved in the candidate ceRNA network. The *x*-axis represents enriched pathways, and the *y*-axis represents −log_10_(*p*-value). GO, Gene Ontology; KEGG, Kyoto Encyclopedia of Genes and Genomes.

Based on the expression profiles of 69 DEmRNAs, 1 DEmiRNA, and 202 DElncRNAs, Pearson correlation analysis was performed to identify potential RNA interaction pairs using the criteria of |correlation coefficient| > 0.80 and *p* < 0.05. Among the 202 candidates of miRNA–lncRNA interaction pairs, 16 negative regulatory relationships were identified (Figure 6A). Likewise, 19 negative regulatory miRNA–mRNA relationships were retained from 79 candidate interaction pairs (Figure 6B). By integrating the DEmRNA, DEmiRNA, and DElncRNA datasets with the ceRNA screening criteria described in Section 4.7, 96 potential lncRNA–miRNA–mRNA interaction relationships were retained and used to construct the candidate ceRNA network (Figure 6C).

To further explore the biological significance of the ceRNA network, functional enrichment analyses were performed on the mRNAs involved in the network. GO enrichment analysis revealed that these mRNAs were significantly enriched in terms such as signaling receptor regulator activity, receptor ligand activity, cell projections, neuronal projections, signal transduction, and neuronal apoptosis. KEGG pathway enrichment analysis showed significant enrichment in the cAMP signaling pathway, cytokine–cytokine receptor interaction, and viral protein interaction with cytokines and cytokine receptor pathways. These findings suggest that the ceRNA network may contribute to hypothalamic functional differences between high- and low-fecundity Xinjiang goats through the regulation of receptor–ligand interactions, cAMP-mediated signal transduction, cytokine-related processes, and neural functions.

Further analysis revealed that all 16 DElncRNAs were associated with candidate lncRNA–chi-miR-141–PCDHB13 regulatory modules. Particularly, the lncRNAs were significantly negatively associated with chi-miR-141, whereas chi-miR-141 was significantly negatively correlated with *PCDHB13*. In contrast, the lncRNAs showed positive correlations with *PCDHB13* expression. The expression correlation patterns and target-prediction results were consistent with the predefined criteria for candidate ceRNA interactions, suggesting that multiple lncRNAs may be involved in the putative regulation of *PCDHB13* through chi-miR-141. Among the 16 lncRNA–chi-miR-141–PCDHB13 candidate modules, five axes showed stronger correlation patterns and statistical significance under the screening criteria described in Section 4.7: MSTRG.4185.16–chi-miR-141–PCDHB13, MSTRG.4335.7–chi-miR-141–PCDHB13, MSTRG.4967.1–chi-miR-141–PCDHB13, MSTRG.18631.16–chi-miR-141–PCDHB13, and MSTRG.25115.21–chi-miR-141–PCDHB13. All five axes fulfilled the target-prediction support and expected ceRNA correlation pattern, including negative correlations between the lncRNAs and chi-miR-141, a negative correlation between chi-miR-141 and *PCDHB13*, and positive correlations between the lncRNAs and *PCDHB13*. Therefore, these axes were retained as key candidate lncRNA–chi-miR-141–PCDHB13 regulatory modules for subsequent discussion. These findings suggest that the lncRNA–chi-miR-141–PCDHB13 axis may represent a potential regulatory module associated with fecundity differences in the hypothalamus of Xinjiang goats.

## 3. Discussion

### 3.1. Differentially Expressed RNAs Reveal Neuroendocrine Signaling and Energy Metabolism Regulatory Features in the Hypothalamus During the Follicular Phase

In the present study, we identified 69 DEmRNAs, 202 DElncRNAs, and 1 DEmiRNA in the follicular-phase hypothalamus of high- and low-fecundity Xinjiang goats. Functional enrichment analysis indicated that the DEmRNAs were primarily associated with the regulation of hormone levels, hormone secretion, peptide hormone secretion, receptor–ligand activity, neuroactive ligand–receptor interactions, and the cAMP signaling pathway. Notably, hormone secretion and receptor–ligand activity are directly involved in signal recognition and transmission along the HPG axis. The pulsatile release of GnRH governs pituitary LH and FSH secretion, thus affecting follicular development, ovulation, and ovarian steroidogenesis [10,11]. Neuroactive ligand–receptor interactions reflect signal transmission between hypothalamic neuropeptides, neurotransmitters, and their receptors, a process closely associated with GnRH neuronal activity, integration of ovarian feedback signals, and reproductive endocrine regulation [10,11,12,13,14]. As an important secondary messenger, cAMP participates in GnRH- and receptor-mediated intracellular signaling and regulates the expression and secretion of gonadotropin-related genes [24]. Therefore, the enrichment patterns of DEmRNAs suggest that the hypothalamus of high- and low-fecundity Xinjiang goats may differ in neuroendocrine signal perception, transduction, and output during the follicular phase.

The predicted target genes of DElncRNAs were mainly enriched in oxidative phosphorylation, the electron transport chain, NADH dehydrogenase complexes, and mitochondrial respiratory chain complexes. Neuronal excitability, synaptic transmission, and neuropeptide secretion in the hypothalamus require a continuous supply of ATP, and are influenced by mitochondrial Ca^2+^ homeostasis and redox balance [25,26,27]. Consequently, the enrichment of DElncRNA target genes in pathways related to mitochondrial energy metabolism may reflect differences in energy supply, neural signaling efficiency, and neuroendocrine regulatory capacity between high- and low-fecundity Xinjiang goats during the follicular phase. Only one significant DEmiRNA was identified in the present study, suggesting that differences in hypothalamic miRNA expression between high- and low-fecundity goats during the follicular phase may not involve widespread alterations, but rather a relatively focused pattern of post-transcriptional regulation. The predicted target genes of this DEmiRNA were enriched in biological processes related to cell adhesion, cell differentiation, nervous system development, and signaling pathways, including MAPK, TGF-β, Hippo, and PI3K-Akt. These pathways are strongly linked to cell proliferation and differentiation, follicular development, granulosa cell function, and steroidogenesis [28,29,30,31,32,33].

Although the sampled tissue in the present study was the hypothalamus rather than the ovary, the enrichment of these pathways among DEmiRNA target genes suggests that miRNA-mediated post-transcriptional regulation may contribute to the modulation of hypothalamic cellular states, neural development, and signal transduction. Notably, the limited sample size, statistical thresholds, and miRNA expression abundance may have influenced the number of DEmiRNAs detected. Therefore, chi-miR-141 should be regarded as a candidate miRNA identified in this study rather than the sole post-transcriptional regulator responsible for fecundity differences in Xinjiang goats.

### 3.2. Analysis of the Chi-miR-141-Associated lncRNA–miRNA–mRNA Candidate Network and Potential Role of PCDHB13

Based on target prediction and expression correlation analyses, a candidate lncRNA–miRNA–mRNA interaction network centered on chi-miR-141 was constructed. Network analysis revealed that 16 lncRNAs potentially interacted with both chi-miR-141 and *PCDHB13*. The predicted target genes associated with these lncRNAs included *NCKAP5*, *PLPP1*, *MTREX*, *EEA1*, *ZNF618*, *ZBTB20*, *DIPC2*, *VAPB*, *RAB22A*, and *MRPS28*. Among these genes, *EEA1* and *RAB22A* are closely involved in endosomal trafficking and membrane protein recycling. The endosomal system regulates the transport and recycling of membrane receptors, neurotrophic factor receptors, and synaptic signaling molecules, thereby influencing neuronal signal transmission [34,35,36]. In hypothalamic neuroendocrine regulation, receptor localization, membrane protein recycling, and maintenance of synaptic signaling may affect neuropeptide secretion and responsiveness to feedback signals. *ZBTB20*, a transcriptional regulator, may play important roles in anterior pituitary development, endocrine cell differentiation, and neural development [37,38], suggesting a potential role in the regulation of hypothalamic–pituitary axis function. Moreover, VAPB is involved in the formation of endoplasmic reticulum–mitochondria contact sites, thereby modulating Ca^2+^ transport, mitochondrial function, and synaptic activity [39,40]. Meanwhile MRPS28 is a mitochondria-related ribosomal protein involved in mitochondrial translation and oxidative phosphorylation [41,42]. In addition, PLPP1 participates in lipid phosphate signaling, and lipid signaling molecules such as lysophosphatidic acid and sphingosine-1-phosphate have been implicated in cell migration, neural development, and reproductive processes [43]. Collectively, the functions of these lncRNA-associated target genes suggest that DElncRNAs may contribute to hypothalamic functional differences between high- and low-fecundity Xinjiang goats by influencing receptor trafficking, neural signaling, endocrine cell function, mitochondrial energy metabolism, and lipid signaling pathways.

Chi-miR-141 was the only significantly DEmiRNA identified in the current study and may therefore represent a focal post-transcriptional regulatory signal in the hypothalamus during the follicular phase. Previous studies in goats have reported elevated expression of miR-141 in small follicles from monotocous goats, suggesting a role in follicular development and granulosa cell function [28]. In granulosa cells, miR-141-3p suppresses apoptosis by targeting DAPK1 and has been implicated in ovarian dysfunction and polycystic ovary syndrome-related processes [44,45]. Moreover, recent studies have demonstrated that miRNAs, lncRNAs, and circRNAs participate in ovarian function and female reproductive disorders by regulating folliculogenesis, steroidogenesis, cell adhesion, oxidative stress, and inflammatory responses [19,20,46,47,48].

*PCDHB13* emerged as a key candidate mRNA node within the predicted regulatory network. All 16 lncRNAs were predicted to form potential interaction modules with *PCDHB13* through chi-miR-141. The lncRNAs were positively correlated with *PCDHB13* expression, whereas chi-miR-141 was negatively correlated with both the lncRNAs and *PCDHB13*. This correlation pattern was consistent with the expected expression profile of a candidate ceRNA relationship. *PCDHB13* belongs to the protocadherin beta family, members of which are important neural cell adhesion molecules within the cadherin superfamily, and are involved in neuronal recognition, neurite development, synapse formation, and neural circuit assembly [49,50,51,52]. Combined with the enrichment of DEmiRNA target genes in processes such as cell adhesion, nervous system development, synaptic function, and neuronal projections, these findings suggest that *PCDHB13* may participate in neuronal connectivity, signal transmission, and neuroendocrine integration within the hypothalamus during the follicular phase. Based on correlation strength and statistical significance, five axes were retained as key candidate regulatory modules: MSTRG.4185.16–chi-miR-141–PCDHB13, MSTRG.4335.7–chi-miR-141–PCDHB13, MSTRG.4967.1–chi-miR-141–PCDHB13, MSTRG.18631.16–chi-miR-141–PCDHB13, and MSTRG.25115.21–chi-miR-141–PCDHB13. Taken together, multiple lncRNAs may form a coordinated regulatory network with chi-miR-141 and PCDHB13, potentially contributing to hypothalamic neural connectivity and the integration of reproductive signaling.

### 3.3. Study Limitations and Future Validation Directions

By integrating mRNA/lncRNA sequencing, small RNA sequencing, expression correlation analysis, target prediction, and RT-qPCR validation, we screened candidate RNA molecules and a putative lncRNA–chi-miR-141–PCDHB13 regulatory module potentially associated with fecundity differences in the hypothalamus of Xinjiang goats during the follicular phase. Nevertheless, because the ceRNA network was primarily constructed based on bioinformatic predictions and expression correlations, it cannot directly demonstrate actual binding interactions, competitive regulatory relationships, or causal links among lncRNAs, chi-miR-141, and *PCDHB13*. In addition, the sequencing sample size was relatively limited. Therefore, the present findings should be interpreted as candidate transcriptomic evidence rather than direct functional validation of a ceRNA-mediated regulatory mechanism. Future studies should include larger sample sizes and integrate individual animal information, serum hormone profiles, binding-site validation assays, protein expression analyses, tissue localization studies, and functional experiments to further verify the biological roles of the candidate RNA modules in fecundity regulation in Xinjiang goats.

## 4. Materials and Methods

### 4.1. Experimental Animals and Sample Collection

The study was performed at a Xinjiang goat breeding farm in Hoboksar Mongol Autonomous County, Tacheng Prefecture, China. All animals were maintained under the same feeding and management conditions throughout the experimental period. The goats were fed a basal diet consisting of roughage and concentrate twice daily and had free access to clean water. Before inclusion in the experiment, all candidate animals were assessed by trained farm personnel and experimental staff. Health status was evaluated based on appetite, behavior, body condition, locomotion, and the absence of obvious clinical signs of respiratory, digestive, parasitic, or reproductive disorders. Only clinically healthy does with normal estrous activity were included.

Healthy Xinjiang does aged 4–5 years with similar body condition and body weight were selected according to kidding records from the previous 2–3 consecutive years. The high-fecundity group comprised eight does that consistently produced twin or multiple offspring, with 2–3 kids per kidding and a mean litter size of 2.2 ± 0.3. The low-fecundity group comprised eight does that consistently produced single offspring, with a mean litter size of 1.0 ± 0.0. These records confirmed a clear and stable fecundity contrast between the two groups.

Estrus synchronization was achieved using intravaginal controlled internal drug release (CIDR) devices (Zoetis, Shanghai, China) for 14 days. Forty-eight hours prior to CIDR removal, pregnant mare serum gonadotropin (PMSG; Ningbo Second Hormone Factory, Ningbo, China) and cloprostenol sodium (PG; Ningbo Second Hormone Factory, Cixi, China) were administered intramuscularly. Estrus onset was monitored using teaser bucks in combination with behavioral observations. Within 48 h of estrus onset, corresponding to the follicular phase, three does per group were randomly selected for euthanasia, tissue collection, and transcriptome sequencing to avoid subjective selection bias. All selected animals met the same criteria for health status, age, body condition, body weight, estrus synchronization response, and reproductive stage. Hypothalamic tissues were rapidly excised, rinsed in sterile 0.9% NaCl, diced, placed in 2 mL cryovials, snap-frozen in liquid nitrogen, and stored at −80 °C until RNA extraction.

### 4.2. RNA Extraction, Quality Assessment, Library Construction, and Sequencing

Total RNA was isolated from hypothalamic tissues using RNAiso Plus (Cat. No. 9109; Takara Bio Inc., Dalian, China) according to the manufacturer’s instructions. RNA concentration and purity were measured using a NanoDrop 2000 spectrophotometer (Thermo Fisher Scientific, Waltham, MA, USA). RNA integrity was initially examined by agarose gel electrophoresis and subsequently assessed with an Agilent 2100 Bioanalyzer (Agilent Technologies, Santa Clara, CA, USA) using the RNA 6000 Nano Kit. Only samples with high integrity were used for library construction. For mRNA and lncRNA libraries, ribosomal RNA was depleted using the Ribo-Zero™ rRNA Removal Kit (Epicentre Biotechnologies, Madison, WI, USA), followed by RNA fragmentation, cDNA synthesis, end repair, adaptor ligation, size selection, and PCR amplification. For miRNA libraries, small RNA (18–30 nt) was ligated to adaptors, reverse transcribed, PCR amplified, and purified by 15% PAGE using the NEBNext Multiplex Small RNA Library Prep Kit (New England Biolabs, Ipswich, MA, USA). Libraries were evaluated for quality and insert size with the Agilent 2100 Bioanalyzer and quantified using PicoGreen fluorescence assays and qPCR. Pooled libraries were sequenced on an Illumina platform (PE150).

### 4.3. Sequencing Data Processing and Alignment

Raw FASTQ files from Illumina sequencing were processed to remove adaptors and low-quality reads using fastp v0.22.0. Reads with quality scores below Q20 were discarded. Clean reads were mapped to the goat reference genome using HISAT2 v2.1.0. For miRNA, reads of 18–36 nt were retained and aligned using miRDeep2 (mapper.pl with Bowtie v2.5.1) for annotation and expression analysis. Unique sequences were used to identify known miRNAs via miRBase, while unannotated reads were used to predict novel miRNAs.

### 4.4. Identification and Differential Expression Analysis of mRNAs, lncRNAs, and miRNAs

Differential expression analyses were conducted as a two-group comparison between high- and low-fecundity Xinjiang goats during the follicular phase, with three biological replicates per group. mRNA expression levels were determined using HTSeq v0.9.1 and subsequently normalized to FPKM. lncRNAs were identified from transcript assemblies generated using StringTie v2.2.3. Candidate transcripts were required to meet the following criteria: transcript length ≥ 200 bp, exon number ≥ 2, coverage > 3, and class codes x, u, or i. Coding potential was evaluated using PLEK v1.2, CNCI v1.0, and PfamScan v1.6.4. Only transcripts classified as non-coding by all three methods were retained as high-confidence lncRNAs. Expression levels were quantified using StringTie and normalized as FPKM values.

Known miRNAs were identified by aligning unique reads against mature and precursor miRNA sequences in the miRBase database. According to the small RNA analysis pipeline, unique reads were mapped to the reference genome using the mapper.pl module of miRDeep2 with Bowtie v2.5.1, whereas reads not annotated as known miRNAs or other small RNA categories were analyzed using MIREAP v0.2 for novel miRNA prediction. To exclude other classes of small RNAs, reads were compared against the piRBase, Rfam, and Repbase databases. Annotation priority was assigned as follows: known miRNA > piRNA > rRNA > tRNA > snRNA > snoRNA > repeat > gene > novel miRNA. Differential expression analyses of mRNAs, lncRNAs, and miRNAs were performed using DESeq v1.39.0. RNAs with |log_2_FoldChange| > 1 and *p* < 0.05 were considered differentially expressed.

### 4.5. Target Gene Prediction and Functional Enrichment Analysis

DEmRNAs were directly analyzed for GO and KEGG enrichment. Target genes of DElncRNAs were predicted using both cis- and trans-regulatory approaches. Cis-target genes were defined as protein-coding genes located within 100 kb upstream or downstream of the lncRNA locus, whereas trans-target genes were identified based on expression correlations between lncRNAs and mRNAs, with thresholds of |correlation coefficient| > 0.95 and *p* < 0.05. DEmiRNA targets were predicted by miRanda v3.3a, with mRNA 3′ untranslated regions serving as target sequences. GO enrichment analysis was performed using topGO v2.50.0, and KEGG pathway enrichment analysis was conducted using clusterProfiler v4.6.0. GO terms or KEGG pathways with *p* < 0.05 were considered significantly enriched.

### 4.6. Comparative and Co-Expression Analyses of lncRNAs and mRNAs

We compared expression levels, chromosomal distribution, transcript length, and exon number between lncRNAs and mRNAs using R v4.5.2 (R Foundation for Statistical Computing, Vienna, Austria). Co-expression relationships were identified by Pearson correlation analysis based on expression profiles across the six biological samples, with significant correlations defined as |correlation coefficient| > 0.95 and *p* < 0.05. Co-expression networks were visualized in Cytoscape v3.10.0. Subsequently, GO and KEGG enrichment analyses were performed on the co-expressed mRNAs, and *p* < 0.05 was considered statistically significant.

### 4.7. Construction of the lncRNA–miRNA–mRNA Regulatory Network

Based on the ceRNA hypothesis, an lncRNA–miRNA–mRNA regulatory network was constructed by integrating DElncRNAs, DEmiRNAs, and DEmRNAs. Potential miRNA–mRNA and miRNA–lncRNA interactions were first identified based on target prediction analyses. These interactions were then integrated with lncRNA–mRNA co-expression results and Pearson correlation analysis across the six biological samples. Candidate miRNA–mRNA and miRNA–lncRNA pairs were retained when they showed negative expression correlations with |correlation coefficient| > 0.80 and *p* < 0.05, whereas candidate lncRNA–mRNA pairs were required to show positive expression correlations consistent with the ceRNA model. Candidate lncRNA–miRNA–mRNA triplets were retained only when the lncRNA, miRNA, and mRNA were differentially expressed, the lncRNA and mRNA shared the same predicted miRNA, the miRNA was negatively correlated with both the lncRNA and mRNA, and the lncRNA was positively correlated with the mRNA. The resulting candidate network was visualized using Cytoscape v3.10.0. Key candidate regulatory axes potentially associated with fecundity in the goat hypothalamus were further screened according to target-prediction support, the expected ceRNA correlation pattern, and correlation strength.

### 4.8. RT-qPCR Validation and Statistical Analysis

To validate the sequencing data, selected DEmRNAs, DElncRNAs, and miRNAs were examined by RT-qPCR using the same six biological samples used for sequencing, including three biological replicates from the high-fecundity group and three biological replicates from the low-fecundity group. For mRNA and lncRNA detection, total RNA was reverse transcribed into cDNA using the All-In-One 5× RT Master Mix (abm, G592). For miRNA analysis, reverse transcription was performed using the miRNA First-Strand cDNA Synthesis Kit (abm, G898). β-actin was used as the internal reference for mRNAs and lncRNAs, and U6 was used as the internal reference for miRNAs. Primers were synthesized by Huazhi Biotechnology Co., Ltd. (Changsha, China), and the primer sequences are listed in Table S9.

RT-qPCR was carried out using SYBR Green chemistry, with three technical replicates for each biological sample. For mRNAs and lncRNAs, the thermal cycling protocol included an initial step of 50 °C for 2 min and 95 °C for 2 min, followed by 40 cycles of 95 °C for 15 s, 60 °C for 15 s, and 72 °C for 30 s. The miRNA protocol consisted of 95 °C for 2 min and 40 cycles of 95 °C for 10 s and 60 °C for 20 s. Relative expression was calculated using the 2^−ΔΔCt^ method. Data are presented as mean ± standard deviation based on three biological replicates per group. Differences between the two groups were analyzed using an independent-samples *t*-test in SPSS 19.0 (SPSS Inc., Chicago, IL, USA), with *p* < 0.05 considered statistically significant.

## 5. Conclusions

In this study, we integrated mRNA/lncRNA transcriptome sequencing and small RNA sequencing to analyze hypothalamic expression profiles in high- and low-fecundity Xinjiang goats during the follicular phase. A total of 69 DEmRNAs, 202 DElncRNAs, and 1 DEmiRNA were identified, and functional enrichment analyses suggested that these RNAs were mainly associated with neuroendocrine signaling, hormone secretion, mitochondrial energy metabolism, and neural functions. The candidate ceRNA network highlighted a putative lncRNA–chi-miR-141–PCDHB13 regulatory module, which may be related to hypothalamic neuronal connectivity and reproductive signal integration. These findings provide candidate RNA molecules and regulatory axes for future functional validation of the molecular mechanisms underlying fecundity differences in Xinjiang goats.

## Data Availability

The raw RNA-seq data generated during this study have been deposited in the NCBI Sequence Read Archive (SRA) under BioProject accession number PRJNA1477308. Additional data supporting the conclusions of this article are available from the corresponding author upon reasonable request.

## Supplementary Materials

The following supporting information can be downloaded at: https://www.mdpi.com/article/10.3390/ijms27167330/s1.

## Abbreviations

The following abbreviations are used in this manuscript:

BP	Biological process
CC	Cellular component
CircRNA	Circular RNA
ceRNA	Competing endogenous RNA
CIDR	Controlled internal drug release
CPM	Counts per million
DEmRNAs	Differentially Expressed messenger RNAs
DEmiRNAs	Differentially Expressed microRNAs
DElncRNAs	Differentially Expressed long non-coding RNAs
FPKM	Fragments per kilobase of transcripts per million mapped reads
FSH	Follicle-stimulating hormone
GnRH	Gonadotropin-releasing hormone
GO	Gene Ontology
HPG	Hypothalamic–pituitary–gonadal
KEGG	Kyoto Encyclopedia of Genes and Genomes
LH	Luteinizing hormone
lncRNA	Long non-coding RNA
MF	Molecular function
MiRNA	MicroRNA
mRNA	Messenger RNA
PCA	Principal component analysis
RT-qPCR	Quantitative real-time PCR
TGF-β	Transforming growth factor-β
XJLDX	High-fecundity group
XJLSX	Low-fecundity group
